# Effect of Natural Variation and Rootstock on Fruit Quality and Volatile Organic Compounds of ‘*Kiyomi tangor*’ (*Citrus reticulata* Blanco) Citrus

**DOI:** 10.3390/ijms242316810

**Published:** 2023-11-27

**Authors:** Tie Wang, Zhendong Zheng, Lijun Deng, Weijia Li, Ya Yuan, Mingfei Zhang, Guochao Sun, Siya He, Jun Wang, Zhihui Wang, Bo Xiong

**Affiliations:** College of Horticulture, Sichuan Agricultural University, Chengdu 611130, China; wangtie@stu.sicau.edu.cn (T.W.); zhengzhendong@stu.sicau.edu.cn (Z.Z.); 2020205017@stu.sicau.edu.cn (L.D.); liweijia@stu.sicau.edu.cn (W.L.); yuanya@stu.sicau.edu.cn (Y.Y.); zhang_mingfei@sicau.edu.cn (M.Z.); 71203@sicau.edu.cn (G.S.); hesiya@sicau.edu.cn (S.H.); 13560@sicau.edu.cn (J.W.); xiongbo1221@sicau.edu.cn (B.X.)

**Keywords:** citrus, mutation, rootstock, volatile organic compounds, fruit quality

## Abstract

In this study, we compared the fruit quality and color of ‘Kiyomi’ (WT) and its mutant (MT) grafted on Ziyang xiangcheng (Cj) (WT/Cj, MT/Cj), and the MT grafted on Trifoliate orange (Pt) (MT/Pt). The differences in sugar, organic acid, flavonoids, phenols, and volatile substances of the three materials were also analyzed by high performance liquid chromatography (HPLC) and headspace solid-phase microextraction-gas chromatography-mass spectrometry (HS-SPME-GC–MS). The results showed significant differences in the appearance of WT/Cj, MT/Cj, and MT/Pt. MT/Pt, compared to WT/Cj, MT/Cj, had lower sugar, acid, phenol and flavonoid contents in the pulp. However, MT/Pt pulp was higher in vitamin C (V_C_), and the peel had significantly higher total phenol and flavonoid contents. In terms of pulp, WT/Cj had the greatest diversity of volatile organic compounds (VOCs). 4-methyl-1-pentanol was significantly higher in MT/Cj pulp, while MT/Pt pulp had a unique octanoic acid, methyl ester. VOCs were more diverse in the peels of the three materials. β-Myrcene and valencen were significantly higher in MT/Cj peels. In contrast, 16 unique VOCs were detected in MT/Pt, and D-limonene content was significantly higher than in WT/Cj and MT/Cj. The results suggest Trifoliate orange is a suitable rootstock for MT.

## 1. Introduction

Citrus belongs to the *Rutaceae* family and is the world’s largest type of fruit. Citrus fruits are rich in bioactive compounds such as vitamin C (V_C_), phenols, carotenoids, folic acid, and dietary fiber, which have beneficial effects on human health by preventing cancer, heart disease, and obesity [1,2,3]. China ranks first among citrus-producing countries in the world, with a planting area of approximately 3 million ha and a yield of approximately 51 million tons in 2021. The abundant germplasm resources play an important role in promoting citrus breeding and the development of the citrus industry.

With the diversification of popular consumption habits, there are new requirements for the quality and variety of citrus. Therefore, the selection and breeding of excellent new citrus varieties has been the focus of industrial development. Citrus is a perennial fruit tree and is polyembryonic, making traditional breeding methods time-consuming and inefficient [4]. In recent years, natural bud mutation has become a major resource for citrus and other fruit tree breeding [5]. Bud variation is the result of somatic mutation, leading to distinctly different phenotypes, also known as bud sports [6]. Previous researchers have reported that bud mutants account for 70% of citrus varieties, such as sweet orange, satsuma mandarin and clementine mandarin [7,8]. Some excellent mutants gain customers’ preference by improving the fruit quality of existing varieties. It has been demonstrated that distinct carotenoid and flavonoid accumulation in a spontaneous mutant of ‘Ponkan’ (*Citrus reticulata* Blanco) resulted in yellowish fruit and enhanced postharvest resistance [9]. The mutation in ‘Fengjiewancheng’ orange affected the sugar and acid metabolism, which ripened one month later than ‘Fengjie 72-1′ navel orange [10]. A better understanding of natural bud mutations is critical to maintain the excellent traits of the cultivars.

Commercial production of citrus is widely dependent on rootstock grafting. The interaction between rootstock and scion affects fruit characteristics. Previous studies on this effect have been extensively documented in many species, including apple [11], pear [12], Japanese plum [13], peach [14], and mango [15]. In the case of citrus, the rootstock affected the external color, peel thickness, juice content, sugars and acids, phenol compounds, and volatile compounds of the fruit [16]. Grafting ‘Or’ and ‘Odem’ mandarins, ‘Valencia’ oranges and ‘Redson’ hybrid on sour orange and Volkamer lemon resulted in total soluble solids and acidity levels of all species being lower in fruit on Volkamer than on sour orange [17]. ‘Lane Late’ sweet orange grafted on six different citrus rootstocks showed significant differences in antioxidant activity and related compounds [18].

The perceived flavor of citrus fruits is determined by a combination of taste and aroma. The sweet and sour flavors are mainly derived from the sugar and acid in the juice capsule. And the aroma of the fruit is provided by a mixture of dozens of volatiles. ‘*Kiyomi tangor*’ (*Citrus unshiu* × *Citrus sinensis* cv. Kiyomi) is the first orange hybrid bred using the Satsuma mandarin (*Citrus unshiu* Marcov.forma miyagawawase) and the Trovita orange (*Citrus sinensis* Osbeck) in Japan [19]. The late ripening characteristic of ‘*Kiyomi tangor*’ fruits holds great promise for their market prospects [20]. The ‘*Kiyomi tangor*’ citrus mutant was discovered in Meishan, Sichuan Province during our field survey. However, little information is known about this mutant. In this work, we compared the differences between ‘Kiyomi’ and its natural mutant at the levels of fruit morphological characteristics, fruit quality, antioxidant capacity, and volatile metabolites. In addition, we found significant differences between the mutant grafted on Trifoliate orange (*Poncirus trifoliata* [L.] Raf.) and Ziyang xiangcheng (*Citrus junos* Sieb. ex Tanaka). Therefore, this study explored the effects on ‘Kiyomi’ fruit from two dimensions: bud variation and rootstock. This result will provide germplasm resources and a theoretical basis for improving the quality of ‘Kiyomi’ fruits.

## 2. Results

### 2.1. Fruit Variation and Color Differences

**Figure 1:**
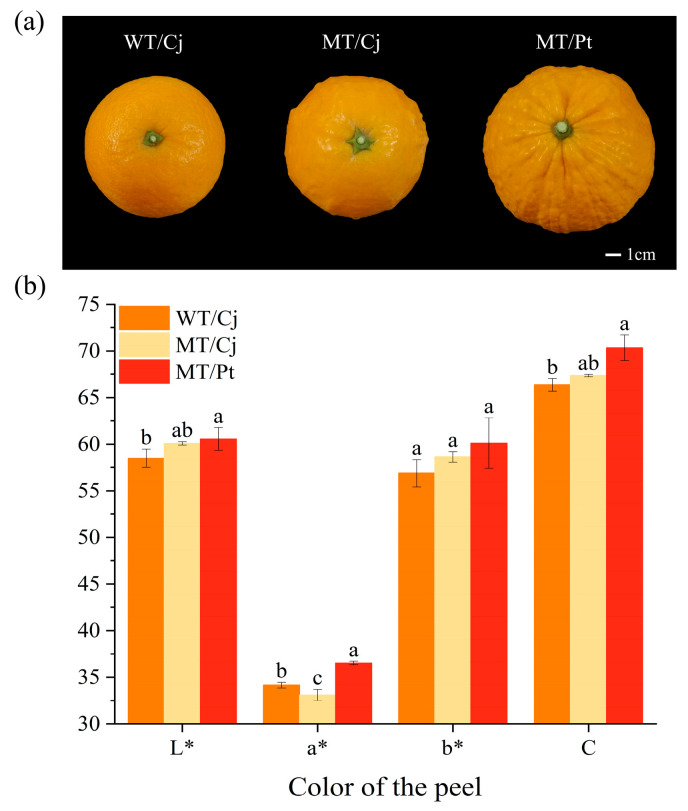
Fruit shape and color differences between ‘*Kiyomi tangor*’ and varieties: (**a**) fruit shape (**b**) color of the peel. Different letters above bars indicate significant differences at the *p* < 0.05 level according to Duncan’s test.

Figure 1a shows the appearance characteristics of the fruit after WT mutation. Compared to WT, the MT peel had a distinctly protuberant character, and the trait was more pronounced especially on grafted Pt rootstocks. In addition, there were significant differences in peel color. Combining the color parameters, we found that MT grafted on Pt had significantly higher L*, a*, and C values, indicating higher brightness, redder color, and higher color purity of MT/Pt peel. In contrast, MTs grafted on Cj rootstocks possessed significantly lower a* values, indicating lower MT/Cj peel color redness [21]. Thus, this result indicates that the mutation gives the MT peel a distinctly different raised character from that of the WT, and that the grafting on different rootstocks exhibits different performance, with the raised character of the MT being more distinct and redder when Pt is used as the rootstock.

### 2.2. Standard Quality Parameters

The effects of different rootstocks on standard quality parameters of MT fruit are shown in Table 1. Compared to WT/Cj and MT/Cj, MT/Pt fruits possessed significantly greater fruit weight, vertical/transverse diameter, and significantly higher V_C_. WT/Cj had significantly higher TSS and TSS/TA. The differences in fruit shape index and TA were not significant among the treatments. These data indicated that both mutation and rootstock treatments had an effect on standard quality parameters of MT fruit.

### 2.3. Sugar and Acid Components

**Figure 2:**
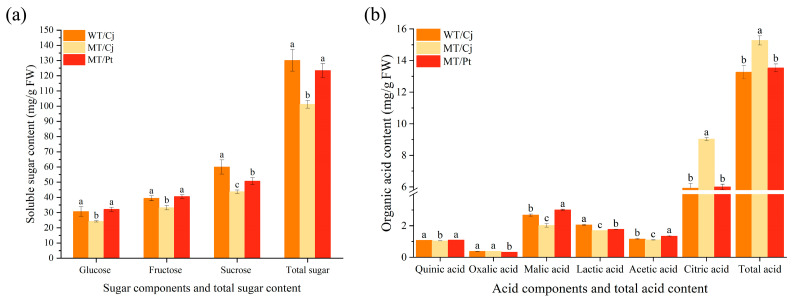
Differences in WT and MT sugar and acid components of different treatments: (**a**) soluble sugar (**b**) organic acids. Different letters above bars indicate significant differences at the *p* < 0.05 level according to Duncan’s test.

Among the sugar components, we found that MT/Cj fruits had significantly lower soluble sugar components content and total content. MT/Pt fruits were not significantly different from WT/Cj treatment in sugar content except for the lower sucrose content (Figure 2a). In the comparison of acid components, we found that Cj rootstock conferred significantly higher levels of oxalic acid, citric acid, and total acid to MT fruits. MT/Pt had significantly higher malic acid and acetic acid content compared to WT/Cj and MT/Cj (Figure 2b).

### 2.4. Phenols Content

#### 2.4.1. Total Flavonoids and Phenols Content

**Figure 3:**
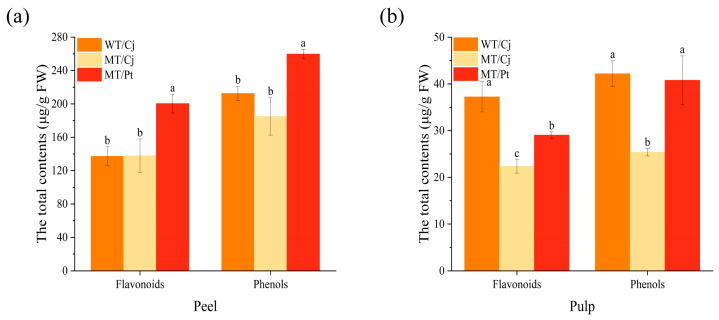
Total flavonoids, phenols in (**a**) peels and (**b**) pulp. Different letters above bars indicate significant differences at the *p* < 0.05 level according to Duncan’s test.

As shown in Figure 3a, WT/Pt peel possessed significantly higher total flavonoids and total phenols content, while the differences between WT/Cj and MT/Cj peel were not significant. In the pulp, total flavonoids content was significantly higher in WT/Cj and lowest in MT/Cj. The difference between WT/Cj and MT/Pt total phenols content was not significant, but MT/Cj content was significantly lower (Figure 3b).

#### 2.4.2. The Contents of Phenol Compounds in Pulp

**Figure 4:**
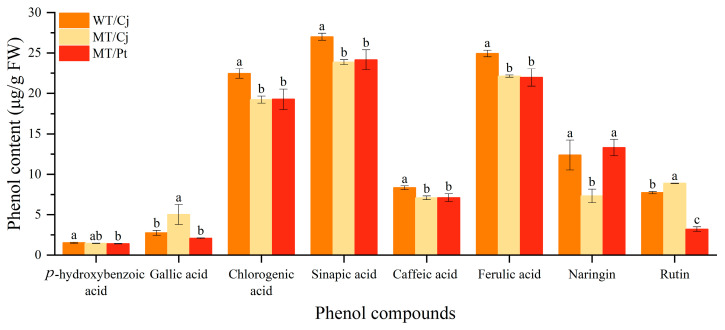
The contents of phenol compounds in pulp. Neohesperidin and naringenin were not detected. Different letters above bars indicate significant differences at the *p* < 0.05 level according to Duncan’s test.

The importance of phenols in human health is well-known, and their potential protective effects are widely recognized [22,23]. Since the pulp is the main edible part of citrus fruit, we conducted an in-depth study using High Performance Liquid Chromatography (HPLC) in order to analyze the reasons for the differences in total pulp phenols between treatments. As shown in Figure 4, a total of eight phenols were detected in the pulp of different treatments. The highest content was sinapic acid and the lowest was *p*-hydroxybenzoic acid. In addition, all WT/Cj components were significantly highest between treatments, except for gallic acid and rutin. In MT fruits, there were effects of different rootstocks on gallic acid, naringin, and rutin contents, and non-significant differences in the other five phenols. Overall, mutation reduced fruit phenol accumulation and different rootstocks also influenced fruit phenol accumulation.

### 2.5. Principal Components Analysis (PCA) and Correlation Analysis

**Figure 5:**
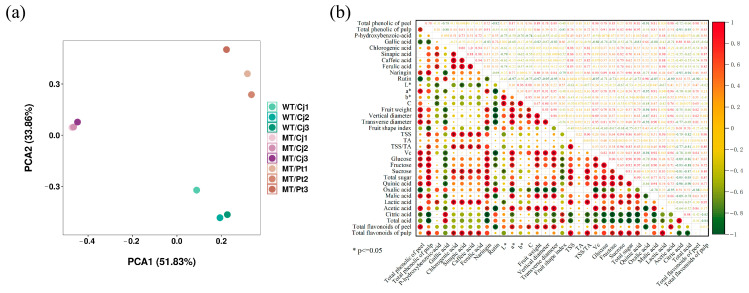
PCA and correlation analysis of the quality parameters of the different treatments: (**a**) PCA analysis. (**b**) correlation analysis.

PCA was used to study the overall variation in the relationship between various quality parameters between treatments [24]. In the present work, the results of the experiment showed significant differences between the treatments (Figure 5). The differences between MT/Cj and WT/Cj treatments with the same rootstock were smaller but significantly larger than MT/Pt. The combined results showed that both mutation and rootstock had an effect on each quality index, but the rootstock effect was significantly more pronounced. In the peel, a* and C showed a significant positive correlation with total flavonoids, indicating that total flavonoids had a significant effect on peel color. In the pulp, we found a positive correlation between TSS and all sugar components, especially sucrose.

### 2.6. VOCs

#### 2.6.1. Identification and Quantification of the VOCs in the Pulp of Different Treatments

**Figure 6:**
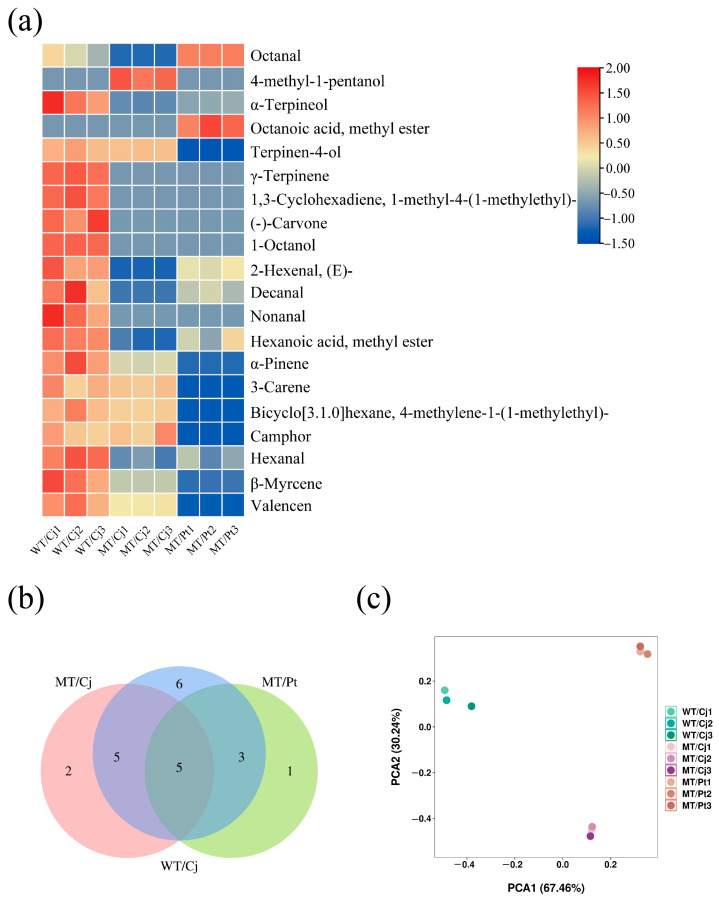
Analysis of the VOCs in the pulp of different treatments: (**a**) heatmap (**b**) Venn diagram (**c**) PCA analysis. D-limonene is not shown in the heatmap.

As shown in Figure 6a,b, the WT/Cj treatment had the highest number of pulp VOCs species and higher content, especially D-limonene (Table S1) and β-Myrcene. The pulp 4-methyl-1-pentanol content was significantly higher in MT/Cj treatment compared to WT/Cj, while MT/Pt had higher octanal. Furthermore, although the MT/Pt treatment pulp had the fewest types and lower levels of VOCs, it contained the unique octanoic acid, methyl ester. PCA analysis showed small material differences between WT/Cj and MT/Cj treatments, but both differed significantly from MT/Pt treatments (Figure 6c).

#### 2.6.2. Identification and Quantification of the VOCs in the Peel of Different Treatments

**Figure 7:**
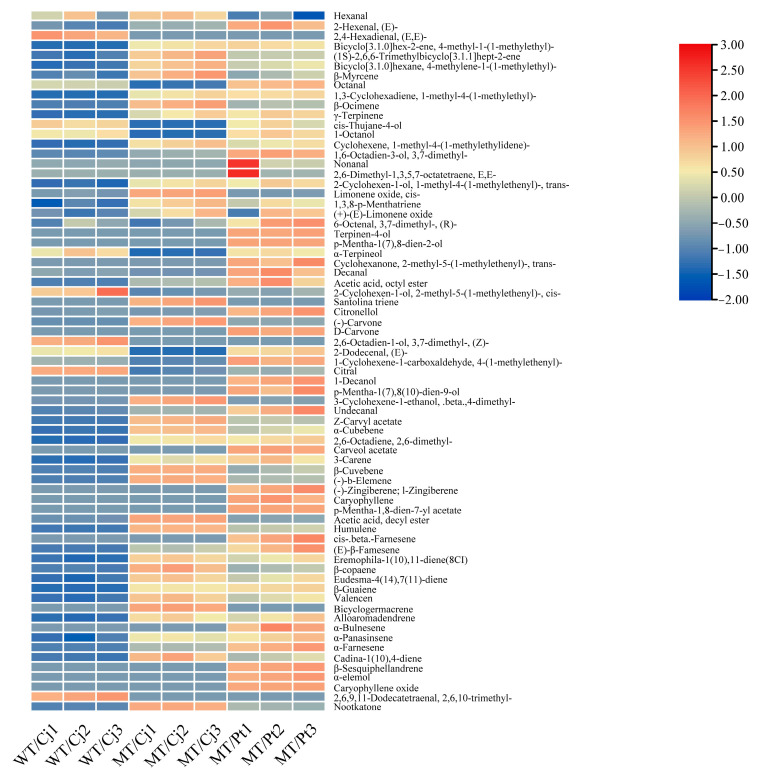
Heatmap of the VOCs in the peel of different treatments. D-limonene is not shown in the heatmap.

**Figure 8:**
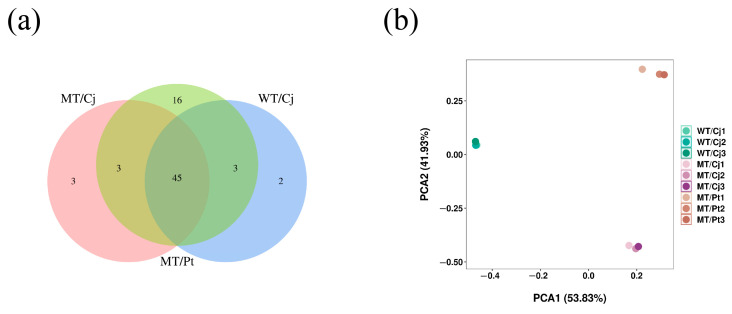
Analysis of the VOCs in the peel of different treatments. (**a**) Venn diagram. (**b**) PCA analysis.

Compared to the pulp, the peel VOCs were significantly more diverse, with 50, 51, and 67 VOCs in the WT/Cj, MT/Cj, and MT/Pt treatments, respectively (Figure 7 and Figure 8). Among them, MT/Pt treatment peel contained unique 16 VOCs such as *p*-mentha-1,8-dien-7-yl acetate, 2,6-dimethyl-1,3,5,7-octatetraene, E, E-, and d-carvone. Overall, the MT/Pt treatment peel had significantly higher VOC content, especially D-limonene, which was 60.65% and 27.06% higher than WT/Cj and MT/Cj, respectively (Table S2). However, MT/Cj contained significantly higher β-Myrcene and valencen. PCA analysis showed large material differences between treatments, especially MT/Pt to WT/Cj, which may imply that rootstocks have a similar function to mutation, both altering the type and content of peel VOCs.

## 3. Discussion

### 3.1. Natural Variation and Rootstocks Effect the General Morphological Characteristics and Nutrient Content of ‘Kiyomi tangor’ Citrus

Citrus is the most produced fruit in the world and provides important nutrients to people [25]. Although cultivars are numerous, they are mainly derived from spontaneous mutations [26]. Our results showed that the mutation conferred a protuberant character to the MT peel. In addition, this trait can become more pronounced by grafting onto Pt rootstocks (Figure 1a). Currently, this unique trait makes MT fruits popular in the market, but the underlying mechanism still needs further study. The more vivid the color of the citrus fruit, the more attention it gets [27]. In the present study, mutation caused a decrease in MT peel redness, but by grafting Pt rootstocks, MT peel redness increased significantly, as evidenced by an increase in a* values. This indicates that the peel color is not only genetically related, but also regulated by the rootstock. This is consistent with the results of previous studies [28,29].

Fruit size and weight are very important economic traits in fruit breeding research [30]. In this study, our results have found significantly smaller weight and size of MT fruits, but significantly larger MT/Pt fruits (Table 1). It is hypothesized that the variation in this trait may be the result of environment, developmental instability, and plasticity [31]. That this variation produces unexpected results is worthy of further study. Previous studies in orange and grape found that the mutants had significantly higher TSS/TA values compared to the wild type, indicating that they would ripen slightly earlier than the parental plants [32,33]. Contrary to this, our results have found that MT fruit TSS/TA was significantly lower, presumably maturing later in MT than WT. In general, the main soluble sugars in most citrus are glucose, fructose, and sucrose, but mainly sucrose. Organic acids, on the other hand, are dominated by citric acid [34,35]. Consistent with this, our results show a similar pattern (Figure 2). Overall, MT/Cj fruits showed low sugar and high acid characteristics, but MT/Pt was not significantly different from WT/Cj. It indicated that rootstocks play an important role in the regulation of sugar-acid metabolism in MT fruits.

### 3.2. Natural Variation and Rootstock Effect the Antioxidant Capacity of ‘Kiyomi tangor’ Citrus Peel and Pulp

Plant polyphenols are phenylpropanoids, including phenol acids, stilbene, curcumin, and flavonoids. These compounds have a wide range of biopharmacological characteristics, such as being antioxidant, anti-inflammatory, and antiviral, and have promising applications [36]. Antioxidant capacity is the link and bridge between multiple biological activities. Citrus flavonoids play a crucial role in the regulation of oxidative stress for health benefits [37]. In this study, compared with WT/Cj, the difference in total flavonoid content of MT/Cj peel was not significant, but MT/Pt peel became significantly higher, presumably the rootstock promoted the accumulation of total flavonoids in MT peel. In contrast, in the pulp, we observed a significantly lower total flavonoid content of MT pulp, speculating that the mutation may have reduced the accumulation of total flavonoids in the pulp. However, it is noteworthy that rootstock (Pt) had a significant effect on the accumulation of total flavonoids in MT pulp (Figure 3). Similar results have been observed in grapes [38] and apples [39]. Among the flavonoid components, we detected only naringin and rutin; neohesperidin and naringenin were not detected (Figure 4). This is in agreement with our previous results [40]. In addition, we also found that the content of flavonoid components and total flavonoid content varied in the same trend between treatments, indicating that our results are reliable.

Citrus peel is a good source of phenols because it is rich in phenol acids [41]. In this study, the total phenols content of MT/Cj and WT/Cj peels were not significantly different, but the total phenol content of MT/Cj pulp was significantly lower. In addition, the MT/Pt peel and pulp total phenol content were significantly higher compared to MT/Cj (Figure 3). This result is consistent with the trend in total flavonoids, which further suggests that mutation may reduce the accumulation of total flavonoids and total phenols in MT pulp, but Pt rootstock has the opposite effect. In the phenol acids, the common phenol acids include caffeic, ferulic, *p*-coumaric, sinapic acids, and so on [42,43]. As shown in Figure 4, we detected a total of 6 phenol acids in the pulp of the fruit. The phenol acid content of the different treatments showed the same pattern, with sinapic acid being the most abundant and *p*-hydroxybenzoic acid being the least abundant. This is consistent with the previous findings in navel orange [44]. In addition, previous authors noted that sinapic acid also increased significantly in Thai fruits during ripening, and they also demonstrated a strong positive correlation between sinapic acid and fruit ripeness [45]. It is speculated that the ripening of ‘*Kiyomi tangor*’ citrus may also be related to sinapic acid.

### 3.3. Natural Variation and Rootstocks Effect Volatile Organic Compounds in the Peel and Pulp of ‘Kiyomi tangor’ Citrus

Today, consumers increasingly prefer foods with raw, natural and delicious flavors, which are closely related to the content of VOCs in food [46]. Our results revealed that the mutation conferred significantly fewer VOC species and total content in MT pulp. Fruits of MT grafted on Pt rootstock had a significantly higher amount of octanal and contained unique octanoic acid and methyl ester, although both VOC species and total content were significantly lower (Figure 6). Previous studies have found that octanal exhibited sweet, citrus, fatty, pungent, green flavors [47] and octanoic acid and methyl ester exhibited exceptional citrus flavors [48]. These unique substances give the MT/Pt pulp its distinctive flavor, which is presumably related to the Pt rootstock. In contrast, VOCs were significantly higher in MT/Cj peels compared to WT/Cj, especially for D-limonene, β-Myrcene, and valencen. In addition, MT/Pt peel showed a similar pattern to MT/Cj and also contained a unique set of 16 VOCs (Table S2, Figure 7 and Figure 8). These results tentatively suggest that the mutation confers significantly higher VOCs content to MT peel and that Pt rootstock has the same effect. This is similar to previous studies on watermelon [49], avocado [50], and grapes [51], indicating that the effect of rootstocks on VOCs is significant.

## 4. Materials and Methods

### 4.1. Plant Materials and Treatment

In this experiment, ‘*Kiyomi tangor*’ (WT) and its mutation (MT) were used as materials. WT grafted on Ziyang xiangcheng (Cj) and Trifoliate orange (Pt) was used as a control (WT/Cj, WT/Pt), and changes in MT fruit grafted on Cj (MT/Cj), and Pt (MT/Pt) were studied. The preceding study conducted by our research group revealed that there was no significant disparity in quality between WT/Cj and WT/Pt fruits at maturity [52]. Therefore, only WT/Cj was selected as the control. Trees were 5 years old and at the same management level in all treatments.

The experiment was conducted with three trees as one treatment, with three replications and a total of 20 healthy fruits collected from each tree from five different positions, with a total of 60 fruits per treatment. Commercially mature fruits were rapidly brought back to the laboratory from Meishan City, Sichuan Province, China. Twelve fruits were randomly selected from each treatment for color determination, and the pulp of the remaining fruits was separated and mixed well, frozen in liquid nitrogen and stored in a −80 °C refrigerator until use.

### 4.2. Color Measurements

The peel color (L*, a*, b*) was measured using a CR-400 (Konica Minolta Inc., Tokyo, Japan). The color purity index C was calculated by the formula: C = (a^2^ + b^2^)^1/2^ [53].

### 4.3. Standard Quality Parameters

Fruit weight was measured by electronic scales and vertical/transverse diameter was measured by vernier calipers. Total soluble solids (TSS) and titratable acids (TA) were measured using a glycolic acid meter (Pocket PAL-BXIACID1; ATAGO, Tokyo, Japan) [54]. V_C_ was measured according to the method described by previous authors [55]. Take 5 mL of juice, dilute with 1% oxalic acid and fix the volume to 50 mL. 5 mL of the diluted solution was pipetted into a 50 mL triangular flask and titrated with 2,6-dichlorophenol indigo dye solution until a stable light pink color was obtained.

### 4.4. Sugar and Acid Compounds

The sugar and acid components of the pulp were measured using high performance liquid chromatography (HPLC). The sugar components were determined and analyzed referring to the previous description [56]. A mixed sample of 2 g was accurately weighed and extracted by adding 4 mL of distilled water. After water bath and centrifugation, the sample was transferred to an injection vial through a 0.45 µm aqueous phase filter membrane to analyze the sugar compositions. Acid components were determined according to our previous method [34,57]. The mixed sample was accurately weighed to 0.5 g, ground with 3 mL of pre-chilled 0.2% metaphosphoric acid, and after volume fixation and centrifugation, the supernatant was taken to determine the organic acid compositions.

### 4.5. Total Flavonoid, Phenol Contents and Phenol Compounds

The total flavonoid contents were determined by a colorimetric method with aluminum chloride [58]. The extract was mixed with 75 μL of 95% ethanol, 10 μL of 10% aluminum chloride, 10 μL of 1.0 M potassium acetate, and 140 μL of distilled water. After incubation, the absorbance was measured at 415 nm. A standard curve was made with rutin, and the data units are μg g^−1^ FW. The Folin–Ciocalteu method was used to quantify the total phenol content, with specific reference to the previous method [59]. The standard curves were made with gallic acid aqueous solution, and the data units are μg g^−1^ FW.

The phenol components were determined by an HPLC method optimized in our laboratory [40]. The mixture of 0.5 g was accurately weighed into a 2 mL centrifuge tube, extracted by adding the extraction solution and protected from light, and the supernatant was used for the analysis of phenol components.

### 4.6. Pulp and Peel Volatile Organic Compounds (VOCs) Identification and Quantification

Headspace solid-phase microextraction-gas chromatography–mass spectrometry (HS-SPME-GC–MS) was used to extract and detect VOCs in different parts of fruits. Fruit tissues (0.1 g peel and 0.5 g pulp) were ground to a powder using liquid nitrogen and quickly placed in a headspace flask, then 5 mL of saturated sodium chloride solution and 50 μL of 1-hexanol (0.1%, *v*/*v*) were added and VOCs were measured after equilibration and extraction [54]. The inlet temperature, desorption time and column chamber warming procedures were performed as previously described [60]. The data were processed using the Data Analysis Application (www.agilent.com/chem, accessed on 30 September 2022), database NIST 11.0/14.0 (NIST/EPA/NIH, Gaithersburg, MD, USA). Combined with NIST data and retention index (RI) data to characterize the substances [61]. The relative quantification of each substance was performed in combination with internal standards [62].

### 4.7. Statistical Analysis

Data were organized using Microsoft Office Excel 2016 (Microsoft, Redmond, Washington, DC, USA), bar graphs were plotted using Origin 2021 (OriginLab Corporation, Northampton, MA, USA), and ANOVA procedure using IBM SPSS 23.0 (SPSS, Inc., Chicago, IL, USA) was used for significance of differences analysis. Heat map, Venn diagram and PCA analysis were performed on the website (https://www.omicstudio.cn/tool, accessed 30 October 2022).

## 5. Conclusions

In conclusion, in terms of fruit appearance quality, the mutation conferred a distinctly protuberant character to the MT peel and a smaller fruit size. When MT was grafted on Pt rootstock, this protuberant character was more pronounced, with significantly larger fruit size and redder peel color. In terms of intrinsic quality, the mutation resulted in significantly lower TSS in MT, but significantly higher V_C_ content in MT/Pt fruit. In terms of antioxidants, the differences in total phenols and total flavonoid contents between WT/Cj and MT/Cj peels were not significant, but the pulp was significantly lower. MT/Pt peel and pulp total phenols and total flavonoid contents were significantly higher than WT/Cj. In terms of VOCs, the mutation reduced the type and content of MT/Cj pulp VOCs, and the MT/Pt treatment was significantly lower. However, in the peel, the differences in the types of MT/Cj peel VOCs were not significant compared to WT/Cj, but the contents were significantly higher, especially for β-Myrcene and valencen. In addition, MT/Pt peel contained a unique set of 16 VOCs. The findings reported here contribute to a better understanding of the differences in fruit quality of ‘*Kiyomi tangor*’ citrus before and after mutation and provide implications for the selection of MT citrus rootstocks.

## Figures and Tables

**Table 1:** The comparison of fruit quality of ‘*Kiyomi tangor*’ variation grafted on Pt and Cj rootstocks.

Treatments	Fruit Weight (g)	Vertical Diameter (mm)	Transverse Diameter (mm)	Fruit Shape Index	TSS (g/100 g FW)	TA (g/100 g FW)	TSS/TA Ratio	V_C_ (g/100 mL FW)
WT/Cj	254.13 ± 4.50 b	72.19 ± 1.93 b	81.82 ± 0.21 b	0.88 ± 0.03 a	10.53 ± 0.25 a	0.82 ± 0.07 a	12.91 ± 1.12 a	28.61 ± 0.47 b
MT/Cj	194.67 ± 16.97 c	70.18 ± 1.49 b	75.31 ± 2.28 c	0.93 ± 0.02 a	7.00 ± 0.10 c	0.80 ± 0.02 a	8.75 ± 0.30 c	23.80 ± 1.23 c
MT/Pt	357.03 ± 11.62 a	81.83 ± 3.34 a	92.34 ± 2.09 a	0.89 ± 0.05 a	8.30 ± 0.17 b	0.81 ± 0.02 a	10.25 ± 0.13 b	35.78 ± 0.49 a

TSS, total soluble solid; TA, titratable acidity. Different letters after the numbers indicate significant differences at the *p* < 0.05 level according to Duncan’s test.

## Data Availability

Data are contained within the article.

## Supplementary Materials

The following supporting information can be downloaded at: https://www.mdpi.com/article/10.3390/ijms242316810/s1.
