# Supplementary material for: Effect of Natural Variation and Rootstock on Fruit Quality and Volatile Organic Compounds of ‘Kiyomi tangor’ (Citrus reticulata Blanco) Citrus
Source: Int J Mol Sci. 2023 Nov 27;24(23):16810. doi: 10.3390/ijms242316810 (PMC10706780; doi:10.3390/ijms242316810)
Supplement: Supplementary file 1 [file ijms-24-16810-s001.zip › Table S1.pdf]

Table S1 Identification and quantification of the VOCs in the pulp of different treatments

**Retention time (minutes)**	**Compound names**	**CAS number**	**Formula**	**RI**	**WT/Cj (μg/g FW)**			**MT/Cj (μg/g FW)**			**MT/Pt (μg/g FW)**		
5.601	Hexanal	000066-25-1	C6H12O	920	5.08	5.41	5.24	3.41	3.56	3.25	3.95	3.36	3.66
7.424	2-Hexenal, (E)-	006728-26-3	C6H10O	1078	0.79	0.60	0.62	0.00	0.00	0.00	0.40	0.37	0.44
8.805	4-methyl-1-pentanol	000626-89-1	C6H14O	1197	0.00	0.00	0.00	2.75	2.41	2.55	0.00	0.00	0.00
10.414	Hexanoic acid, methyl ester	000106-70-7	C7H14O2	1029	1.05	1.02	0.99	0.51	0.45	0.45	0.73	0.61	0.84
10.58	α-Pinene	000080-56-8	C10H16	949	1.09	1.37	1.02	0.58	0.56	0.59	0.00	0.00	0.00
12.495	Bicyclo[3.1.0]hexane, 4-methylene-1-(1-methylethyl)-	003387-41-5	C10H16	979	0.63	0.76	0.60	0.55	0.55	0.56	0.00	0.00	0.00
13.474	β-Myrcene	000123-35-3	C10H16	994	7.30	6.50	5.32	3.04	3.02	3.07	0.61	0.69	0.77
14.094	Octanal	000124-13-0	C8H16O	1033	4.78	3.63	2.48	0.00	0.00	0.00	6.99	7.04	7.09
14.709	1,3-Cyclohexadiene, 1-methyl-4-(1-methylethyl)-	000099-86-5	C10H16	1013	0.21	0.23	0.20	0.00	0.00	0.00	0.00	0.00	0.00
15.447	D-Limonene	005989-27-5	C10H16	1024	503.73	526.69	440.25	235.71	238.82	239.30	57.27	49.12	65.41
17.293	γ-Terpinene	000099-85-4	C10H16	1052	0.32	0.33	0.31	0.00	0.00	0.00	0.00	0.00	0.00
18.411	1-Octanol	000111-87-5	C8H18O	1148	0.94	0.95	0.92	0.00	0.00	0.00	0.00	0.00	0.00
20.705	Nonanal	000124-19-6	C9H18O	1141	1.94	1.55	1.16	0.00	0.00	0.00	0.00	0.00	0.00
22.481	Octanoic acid, methyl ester	000111-11-5	C9H18O2	1178	0.00	0.00	0.00	0.00	0.00	0.00	1.24	1.59	1.41
23.486	Camphor	000076-22-2	C10H16O	1147	0.75	0.63	0.61	0.65	0.61	0.81	0.00	0.00	0.00
26.583	Terpinen-4-ol	000562-74-3	C10H18O	1195	0.33	0.36	0.31	0.31	0.32	0.31	0.00	0.00	0.00
27.899	α-Terpineol	000098-55-5	C10H18O	1215	3.08	2.50	2.17	0.49	0.43	0.47	0.75	0.80	0.84
29.712	Decanal	000112-31-2	C10H20O	1243	0.77	0.97	0.56	0.00	0.00	0.00	0.30	0.35	0.25

33.178	(-)-Carvone	006485-40-1	C10H14O	1296	0.20	0.17	0.23	0.00	0.00	0.00	0.00	0.00	0.00
47.347	3-Carene	013466-78-9	C14H24O<br>2	1364	0.99	0.74	0.86	0.78	0.75	0.80	0.00	0.00	0.00
60.831	Valencen	004630-07-3	C15H24	1477	5.05	5.67	4.42	3.39	3.45	3.51	0.00	0.00	0.00
