# Supplementary material for: Effect of Natural Variation and Rootstock on Fruit Quality and Volatile Organic Compounds of ‘Kiyomi tangor’ (Citrus reticulata Blanco) Citrus
Source: Int J Mol Sci. 2023 Nov 27;24(23):16810. doi: 10.3390/ijms242316810 (PMC10706780; doi:10.3390/ijms242316810)
Supplement: Supplementary file 1 [file ijms-24-16810-s001.zip › Table S2.pdf]

Table S2 Identification and quantification of the VOCs in the peel of different treatments

Retention time (minutes)	Compound names	CAS number	Formula	RI	WT/Cj (μg/g FW)			MT/Cj (μg/g FW)			MT/Pt (μg/g FW)		
5.547	Hexanal	000066-25-1	C6H12O	915	716.53	784.50	648.56	777.01	788.72	765.29	611.43	656.98	565.87
7.328	2-Hexenal, (E)-	006728-26-3	C6H10O	1069	619.45	582.56	563.54	673.71	666.28	681.14	906.40	939.10	873.70
9.767	2,4-Hexadienal, (E,E)-	000142-83-6	C6H8O	1280	21.94	20.03	18.12	0.00	0.00	0.00	0.00	0.00	0.00
10.388	Bicyclo[3.1.0]hex-2-ene, 4-methyl-1-(1-methylethyl)-	028634-89-1	C10H16	946	0.00	0.00	0.00	16.36	17.57	18.77	19.16	18.36	17.56
10.629	(1S)-2,6,6-Trimethylbicyclo[3.1.1]hept-2-ene	007785-26-4	C10H16	950	225.06	203.06	247.06	539.31	574.11	608.91	408.01	413.40	418.79
12.549	Bicyclo[3.1.0]hexane, 4-methylene-1-(1-methylethyl)-	003387-41-5	C10H16	979	152.60	172.54	192.47	661.54	708.02	754.50	490.91	534.22	577.53
13.586	β-Myrcene	000123-35-3	C10H16	995	1774.76	1878.43	1671.08	3023.30	3183.32	3343.34	2100.43	2298.73	2497.03
14.223	Octanal	000124-13-0	C8H16O	1037	663.05	652.51	641.98	358.13	375.24	392.35	818.18	833.95	849.73
14.843	1,3-Cyclohexadiene, 1-methyl-4-(1-methylethyl)-	000099-86-5	C10H16	1015	0.00	0.00	0.00	34.05	37.74	41.43	38.53	39.68	40.83
16.159	D-Limonene	005989-27-5	C10H16	1035	71850.59	70018.95	68187.30	94580.46	88527.31	82474.16	112482.80	118701.42	106264.17
16.924	β-Ocimene	013877-91-3	C10H16	1047	25.81	25.09	26.52	290.52	311.67	332.82	124.55	148.71	145.01
17.459	γ-Terpinene	000099-85-4	C10H16	105	12.37	12.30	12.44	28.14	31.32	34.50	31.69	34.75	33.73

				5									
17.956	cis-Thujane-4-ol	015537-55-0	$C_{10}H_{18}O$	1062	12.85	11.74	12.30	0.00	0.00	0.00	10.81	12.50	9.12
18.432	1-Octanol	000111-87-5	$C_8H_{18}O$	1148	72.90	70.23	75.57	22.13	22.68	23.22	75.42	82.54	77.53
19.374	Cyclohexene, 1-methyl-4-(1-methylethylidene)-	000586-62-9	$C_{10}H_{16}$	1084	28.31	27.83	28.80	66.72	70.47	74.22	59.43	63.49	67.55
20.459	1,6-Octadien-3-ol, 3,7-dimethyl-	000078-70-6	$C_{10}H_{18}O$	1101	425.78	417.12	434.45	533.54	552.25	570.97	911.83	930.30	893.36
20.754	Nonanal	000124-19-6	$C_9H_{18}O$	1142	99.64	91.84	107.44	89.74	93.05	96.36	911.83	265.55	250.58
21.058	2,6-Dimethyl-1,3,5,7-octatetraene, E,E-	000460-01-5	$C_{10}H_{14}$	1110	0.00	0.00	0.00	0.00	0.00	0.00	911.83	15.06	14.96
21.797	2-Cyclohexen-1-ol, 1-methyl-4-(1-methylethenyl)-, trans-	007212-40-0	$C_{10}H_{16}O$	1121	22.11	22.97	21.25	38.78	40.48	42.17	39.53	43.35	41.52
22.823	Limonene oxide, cis-	013837-75-7	$C_{10}H_{16}O$	1137	8.16	8.49	7.83	17.80	17.99	18.18	8.11	8.02	8.30
22.984	1,3,8-p-Menthatriene	18368-95-1	$C_{10}H_{14}$	1139	20.64	23.03	21.83	28.90	29.84	30.77	26.76	29.20	28.25
23.193	(+)-(E)-Limonene oxide	006909-30-4	$C_{10}H_{16}O$	1143	95.21	90.62	92.91	105.36	110.11	114.87	91.74	114.87	112.69
24.738	6-Octenal, 3,7-dimethyl-, (R)-	002385-77-5	$C_{10}H_{18}O$	1166	80.52	93.50	87.01	78.38	84.35	90.32	100.27	110.48	112.11
26.562	Terpinen-4-ol	000562-74-3	$C_{10}H_{18}O$	1194	0.00	0.00	0.00	0.00	0.00	0.00	86.34	84.56	88.12
27.632	*p*-Mentha-1(7),8-dien-2-ol	035907-10-9	$C_{10}H_{16}$	121	0.00	0.00	0.00	0.00	0.00	0.00	42.87	43.02	42.73

			O	1									
27.926	α-Terpineol	000098-55-5	C10H18 O	121 5	316.93	368.68	342.81	141.77	147.75	153.73	333.67	344.58	322.76
28.541	Cyclohexanone, 2-methyl-5-(1-methylethenyl)-, trans-	005948-04-9	C10H16 O	122 5	0.00	0.00	0.00	0.00	0.00	0.00	45.20	38.10	52.31
29.793	Decanal	000112-31-2	C10H20 O	124 4	490.23	420.30	455.27	344.69	347.79	350.90	1409.30	1562.21	1256.39
30.595	Acetic acid, octyl ester	000112-14-1	C10H20 O2	125 6	0.00	0.00	0.00	50.36	52.46	54.55	130.90	153.76	108.04
30.755	2-Cyclohexen-1-ol, 2-methyl-5-(1-methylethenyl)-, cis-	001197-06-4	C10H16 O	125 9	116.15	116.91	142.13	70.75	74.12	77.50	83.69	80.20	87.19
31.092	Santolina triene	002153-66-4	C10H16	126 4	0.00	0.00	0.00	34.51	37.64	40.78	0.00	0.00	0.00
32.168	Citronellol	000106-22-9	C10H20 O	128 0	51.97	54.75	57.53	53.17	55.29	57.41	142.05	151.99	161.93
32.151	(-)-Carvone	000106-22-9	C10H20 O	128 0	0.00	0.00	0.00	259.41	275.02	290.63	44.60	43.01	46.19
33.098	D-Carvone	002244-16-8	C10H14 O	129 5	0.00	0.00	0.00	0.00	0.00	0.00	288.80	269.17	278.99
34.885	2,6-Octadien-1-ol, 3,7-dimethyl-, (Z)-	000106-25-2	C10H18 O	132 2	18.82	19.41	21.97	0.00	0.00	0.00	0.00	0.00	0.00
35.403	2-Dodecenal, (E)-	020407-84-5	C12H22 O	125 7	59.25	62.50	65.75	0.00	0.00	0.00	67.47	69.33	75.87
36.142	1-Cyclohexene-1-carboxaldehyde, 4-(1-methylethenyl)-	002111-75-3	C10H14 O	134 1	230.77	225.95	221.14	148.20	157.91	167.61	418.13	393.47	405.80
36.404	Citral	005392-40-5	C10H16	134	313.25	314.40	315.56	166.70	177.71	188.72	217.24	211.33	223.15

			O	5									
36.874	1-Decanol	000112-30-1	C10H22O	1352	0.00	0.00	0.00	0.00	0.00	0.00	24.52	27.09	29.45
38.243	*p*-Mentha-1(7),8(10)-dien-9-ol	029548-13-8	C10H16O	1373	0.00	0.00	0.00	0.00	0.00	0.00	112.59	95.90	129.29
38.869	3-Cyclohexene-1-ethanol, .beta.,4-dimethyl-	018479-68-0	C10H18O	1383	12.44	12.82	13.20	148.50	157.52	166.54	20.32	24.93	22.62
40.543	Undecanal	000112-44-7	C11H22O	1327	20.41	21.68	22.95	34.50	34.79	35.08	57.56	65.39	73.21
43.897	Z-Carvyl acetate	001205-42-1	C12H18O2	1336	9.14	8.88	9.41	76.14	79.29	82.44	43.46	45.33	41.59
44.657	α-Cubebene	017699-14-8	C15H24	1329	14.64	15.62	15.13	44.23	44.71	45.19	30.32	33.70	37.08
46.047	2,6-Octadiene, 2,6-dimethyl-	002792-39-4	C10H18	1493	23.58	23.02	24.14	76.91	80.07	83.23	78.85	83.77	88.70
46.694	Carveol acetate	000097-42-7	C12H18O2	1363	0.00	0.00	0.00	0.00	0.00	0.00	36.84	37.69	36.00
47.395	3-Carene	013466-78-9	C10H16	1514	420.71	402.23	439.19	1305.21	1217.28	1393.15	1493.74	1626.65	1360.82
49.246	β cubebene	013744-15-5	C15H24	1371	40.00	40.93	39.06	123.97	123.28	124.66	62.54	71.52	80.51
49.577	(-)-b-Elemene	000515-13-9	C15H24	1374	149.81	143.98	155.64	1259.71	1275.99	1243.44	548.59	583.04	617.49
51.583	(-)-Zingiberene; l-Zingiberene	000495-60-3	C15H24	1393	0.00	0.00	0.00	0.00	0.00	0.00	13.65	15.89	18.12
52.145	Caryophyllene	000087-44-5	C15H24	139	0.00	0.00	0.00	0.00	0.00	0.00	447.04	488.56	405.52

				8									
52.289	*p*-Mentha-1,8-dien-7-yl acetate	015111-96-3	C12H18O2	1415	0.00	0.00	0.00	0.00	0.00	0.00	523.31	523.27	523.23
52.877	Acetic acid, decyl ester	000112-17-4	C12H24O2	1420	8.77	8.53	9.02	253.79	252.51	255.06	34.88	39.32	43.77
56.001	Humulene	006753-98-6	C15H24	1433	40.28	39.16	41.40	170.91	171.61	170.22	103.22	109.29	115.36
57.317	cis-β-Farnesene	028973-97-9	C15H24	1445	0.00	0.00	0.00	0.00	0.00	0.00	21.37	25.67	29.97
57.894	(E)-β-Famesene	018794-84-8	C15H24	1450	52.71	55.13	50.28	279.58	253.91	305.24	435.69	519.64	603.58
58.568	Eremophila-1(10),11-diene(8CI)	010219-75-7	C15H24	1456	14.80	13.46	14.13	30.24	30.94	29.54	25.27	27.48	29.70
59.381	β-copaene	1000374-18-9	C15H24	1464	21.19	22.15	20.24	61.36	64.63	58.09	33.89	37.22	40.55
59.713	Eudesma-4(14),7(11)-diene	000515-17-3	C15H24	1467	66.48	62.07	70.88	140.76	144.72	136.80	111.79	123.64	135.48
60.51	β-Guaiene	000088-84-6	C15H24	1474	26.91	26.78	27.03	45.15	45.31	44.99	46.13	46.71	47.29
61.039	Valencen	004630-07-3	C15H24	1479	1023.17	957.58	1088.75	2550.99	2648.69	2453.29	1890.13	2094.74	2299.34
61.216	Bicyclogermacrene	067650-90-2	C15H24	1481	0.00	0.00	0.00	42.10	43.27	40.93	0.00	0.00	0.00
62.061	Alloaromadendrene	025246-27-9	C15H24	1488	20.95	20.23	21.67	44.83	46.95	42.71	38.92	43.45	47.98
62.462	α-Bulnesene	003691-11-0	C15H25	149	0.00	0.00	0.00	0.00	0.00	0.00	19.53	27.83	23.68

				2									
63.436	(-)-α-Panasinsen	056633-28-4	$C_{15}H_{24}$	1501	67.22	60.86	73.58	113.62	116.59	110.65	116.16	122.86	129.56
63.896	α-Farnesene	000502-61-4	$C_{15}H_{24}$	1505	14.39	12.29	13.34	218.23	211.06	225.40	495.27	546.34	597.41
64.767	Cadina-1(10),4-diene	000483-76-1	$C_{15}H_{24}$	1513	68.37	65.22	62.07	212.28	227.71	196.84	126.47	144.05	161.63
65.062	β-Sesquiphellandrene	020307-83-9	$C_{15}H_{25}$	1516	0.00	0.00	0.00	0.00	0.00	0.00	22.58	23.96	25.34
67.677	α-elemol	000639-99-6	$C_{15}H_{26}$	1540	0.00	0.00	0.00	0.00	0.00	0.00	12.80	13.67	14.54
70.726	Caryophyllene oxide	001139-30-6	$C_{15}H_{27}$	1567	0.00	0.00	0.00	0.00	0.00	0.00	38.52	39.26	40.01
77.278	2,6,9,11-Dodecatetraenal, 2,6,10-trimethyl-	004955-32-2	$C_{15}H_{22}O$	1627	6.13	6.60	7.07	0.00	0.00	0.00	0.00	0.00	0.00
79.08	Nootkatone	004674-50-4	$C_{15}H_{22}O$	1644	5.47	5.76	6.04	24.89	25.23	24.56	12.47	11.78	11.09
